# A Comparison of the Health Effects of Ambient Particulate Matter Air Pollution from Five Emission Sources

**DOI:** 10.3390/ijerph15061206

**Published:** 2018-06-08

**Authors:** Neil J. Hime, Guy B. Marks, Christine T. Cowie

**Affiliations:** 1 Woolcock Institute of Medical Research, University of Sydney, 431 Glebe Point Road, Glebe, Sydney, NSW 2037, Australia; guy.marks@sydney.edu.au (G.B.M.); christine.cowie@sydney.edu.au (C.T.C.); 2 The Sydney School of Public Health, University of Sydney Medical School, Sydney, NSW 2006, Australia; 3 South West Sydney Clinical School, University of New South Wales, Goulburn Street, Liverpool, Sydney, NSW 2170, Australia; 4 Ingham Institute of Applied Medical Research, 1 Campbell Street, Liverpool, Sydney, NSW 2170, Australia

**Keywords:** air pollution, particulate matter, source-specific, health effects

## Abstract

This article briefly reviews evidence of health effects associated with exposure to particulate matter (PM) air pollution from five common outdoor emission sources: traffic, coal-fired power stations, diesel exhaust, domestic wood combustion heaters, and crustal dust. The principal purpose of this review is to compare the evidence of health effects associated with these different sources with a view to answering the question: Is exposure to PM from some emission sources associated with worse health outcomes than exposure to PM from other sources? Answering this question will help inform development of air pollution regulations and environmental policy that maximises health benefits. Understanding the health effects of exposure to components of PM and source-specific PM are active fields of investigation. However, the different methods that have been used in epidemiological studies, along with the differences in populations, emission sources, and ambient air pollution mixtures between studies, make the comparison of results between studies problematic. While there is some evidence that PM from traffic and coal-fired power station emissions may elicit greater health effects compared to PM from other sources, overall the evidence to date does not indicate a clear ‘hierarchy’ of harmfulness for PM from different emission sources. Further investigations of the health effects of source-specific PM with more advanced approaches to exposure modeling, measurement, and statistics, are required before changing the current public health protection approach of minimising exposure to total PM mass.

## 1. Health Effects of Particulate Matter (PM) Air Pollution

### 1.1. Observations of the Health Effects of PM Air Pollution Particle Mass

The Global Burden of Diseases Study 2015 has estimated the global extent of the health burden of exposure to ambient PM air pollution, with 7.6% of global deaths and 4.2% of global disability-adjusted life years in 2015 attributable to exposure to PM with aerodynamic diameter less than 2.5 µm (PM_2.5_) [1]. Assessments of the disease burden of exposure to PM air pollution are based on the results of large cohort studies that have found associations between mass concentrations of ambient PM air pollution and adverse health outcomes. The long running *Harvard Six Cities Study* [2,3] and *American Cancer Society Cancer Prevention Study II* [4,5], the latter an ongoing study of over one million adults, are among many large, multicentre studies that have observed statistically significant associations between long-term exposure to PM air pollution and deaths [6,7,8].

Exposure to PM air pollution has been associated with a range of cardiovascular and respiratory disease endpoints. Observations from cohorts participating in the multi-country *European Study of Cohorts for Air Pollution Effects* (ESCAPE) project show that long-term exposure to PM air pollution is associated with the incidence of acute coronary events (myocardial infarction and unstable angina) [9] and stroke [10]. Other investigations within ESCAPE have found long-term exposure to PM air pollution to be associated with decreased lung function in children [11] and adults [12] (a finding also observed in the *Framingham Heart Study* cohort in the north eastern United States [13]), the prevalence and incidence of chronic obstructive pulmonary disease (COPD) [14], and childhood pneumonia [15]. Data from three other European cohorts suggest that long-term exposure to PM may be associated with asthma prevalence [16]. Among other health effects found to be associated with exposure to PM air pollution in the ESCAPE cohorts are lung cancer incidence [17] and low birth weight [18].

Large collaborative studies of the associations between daily PM air pollution and mortality, the *Air Pollution and Health: A European and North American Approach* (APHENA) and the MED-PARTICLES project in Mediterranean Europe, have shown short-term exposure to PM to be associated with all-cause, cardiovascular, and respiratory mortality [19,20,21]. Daily mortality data from the National Center for Health Statistics has been utilised to demonstrate associations between daily PM air pollution and mortality in studies of 75 and 112 US cities [19,22]. A meta-analysis of short-term exposure studies found that short-term exposure to PM_2.5_ was more strongly associated with respiratory causes of death than for cardiovascular causes [23].

The MED-PARTICLES project has shown daily PM to be associated with hospital admissions for both cardiovascular and respiratory disease [24]. A meta-analysis of data from 36 panel studies found short-term exposure to PM air pollution to be associated with episodes of asthma symptoms in asthmatic children [25]. Other health effects found to be associated with exposure to PM air pollution include exacerbations of COPD, impaired vascular function, high blood pressure, stroke, myocardial infarction and neurological diseases such as Alzheimer’s and Parkinson’s disease [26].

Epidemiological studies of PM air pollution have generally associated adverse health effects with exposure to particle mass concentration. However, not all particles are the same. Ambient PM is a heterogeneous mix of particles with different physical and chemical characteristics. Particles collected at different sites have different characteristics that are largely dependent on the types of emission sources present [27], and seasonality is a strong contributor to PM variability. It is likely that the various physical and chemical characteristics of particles contribute to their capacity to affect health [28,29,30,31]. For example, the sulphate, iron, nickel, aluminium, and zinc content of PM_2.5_ have specifically been associated with daily mortality [19,32,33,34]. It is important to note that natural sources of PM such as crustal dust and sea salt contribute to the total ambient PM, especially in natural settings but also in urban areas.

The current policy approach to managing PM air pollution is to set guideline limits for the ambient mass concentration of PM. Regulation of PM emissions aims to keep ambient PM air pollution below those guideline limits and therefore the mass of particles that is emitted is considered more important than the type of source-specific PM. If different particle characteristics elicit varying impacts on health, and PM from various emission sources have different characteristics, then better understanding of the relative health impacts of PM from different sources will enable development of public policy that targets specific emission sources to maximise benefits to human health.

### 1.2. Towards an Understanding of the Health Effects Specific to PM Air Pollution from Different Sources

In recent years there has been increasing effort to understand the nature of the health impacts of different types of particles that originate from different emission sources. Some investigations have provided insight into which components of PM may be responsible for particle toxicity and human health effects. The Health Effects Institute funded *National Particle Component Toxicity* (NPACT) initiative is one such investigation [35]. The ESCAPE project has also investigated the health effects of PM constituents and together with the *Transport related Air Pollution and Health impacts—Integrated Methodologies for Assessing Particulate Matter* (TRANSPHORM) project has used land use regression to estimate exposure to PM constituents [36,37]. In this review we discuss the findings of studies, within and outside of these projects, which have examined the health effects associated with exposure to source-specific PM. Although there are other PM emission sources with important health impacts, this review focuses on sources for which there is considerable population exposure, and studies of the comparative effects of PM from different sources, namely traffic, coal-fired power stations, diesel exhaust, domestic wood combustion heaters, and crustal dust.

A key question is not only whether exposure to PM from a specific source is associated with adverse health effects, but whether exposure to PM from some emission sources is associated with worse health outcomes than equivalent exposure to PM from other sources. An understanding of any differences in the health impact of PM air pollution from different sources will inform environmental policy and air pollution emissions regulation that aims to maximize health benefits. To this end, this review of evidence of the health effects of source-specific PM air pollution has a particular focus on studies that have compared the health effects of exposure to PM from different emission sources in multi-pollutant models.

This article is not intended to be a comprehensive review of all of the evidence of health effects associated with PM from the five different emission sources that are considered. Rather, the article provides brief reviews of the health effects associated with PM from different emissions sources and an overview of the findings of comparative studies of the health effects of PM from different sources. The methods employed in those studies are described and briefly discussed, however an in-depth interrogation of the pros and cons of various methods is outside the scope of this article. This is not a systematic review as per *Preferred Reporting Items for Systematic Reviews and Meta-Analyses* (PRISMA). However, prior to drafting the manuscript, keyword searches were conducted of the scientific databases Web of Science^TM^ and PubMed for the years 1966 to 2016. Relevant publications were identified from a review of article titles and abstracts. The grey literature, including government agency and non-government research institute websites, was also searched.

The following inclusion/exclusion criteria were applied to the literature search:

Inclusion criteria

- Epidemiological studies that gave due consideration to measured and unmeasured confounders of the exposure–health response relationship
- Controlled exposure studies in humans or animals, where exposure was by way of inhalation of concentrated or ambient levels of air pollution particles
- Reviews of scientific evidence

Exclusion criteria

- Air pollution exposure studies and source speciation data without health outcome data
- Epidemiological studies that did not consider PM air pollution from at least one of the five sources examined in this review
- Epidemiological studies in which the study cohort and/or pollution exposure data was deemed too small to have external validity
- Quantitative studies that did not characterize the uncertainty of effect estimates (i.e., lacking confidence intervals or standard errors)
- Studies of the effects of gas/particle mixtures without consideration of the effects of the PM component were generally excluded
- Cell culture or molecular studies that provided no mechanistic insight into health effects observed in epidemiological studies

**Figure 1:**
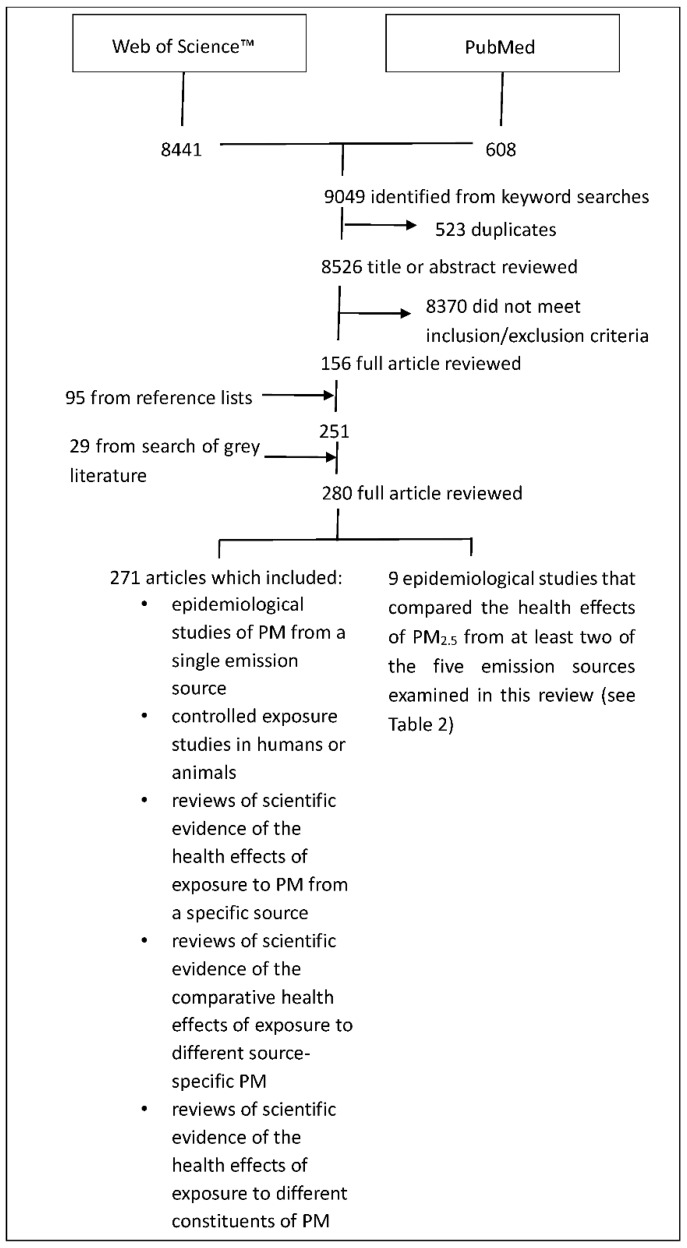
Results of search of scientific databases.

A total of 8526 different article titles were identified from keyword searches of the scientific databases (Figure 1). Of those articles, 8370 did not meet the inclusion/exclusion criteria, leaving 156 articles for full review. In addition, 95 further articles were obtained from the reference lists of the 156 articles and 29 publications were obtained from grey literature, resulting in a total of 280 articles that were examined in full.

Most of the articles that were examined for this review did not compare the health effects associated with PM air pollution from different sources, but instead were studies of the health effects associated with PM from a single emission source. For those epidemiological studies that investigated health outcomes associated with PM from at least two of the five emission sources examined in this review, we quantitatively compare the relative risks associated with an increase in exposure to the different source-specific PM. There have been previous reviews of the comparative differences in the health effects of different components and sources of PM. The conclusions from those reviews are summarized below in Table 1. This list does not include reviews that only examined PM from a single source such as traffic.

From previous reviews of the differences in the health effects of PM from different sources it is unclear if there is a hierarchy in the harmfulness of PM from different sources. Therefore, this current review was undertaken with the following objectives:

1. Review the evidence of the health effects associated with PM air pollution from traffic, coal-fired power stations, diesel exhaust, domestic wood combustion heaters, and crustal dust and qualitatively compare the weight of evidence of health effects associated with PM from those emission sources. Both investigations that examined the health effects associated with PM from a single source and investigations that compared health effects from PM from different sources were included in this review of evidence.
2. Conduct a quantitative comparison of the health effects of source-specific PM using epidemiological studies that compared the health effects associated with PM from different sources within the same study.
3. Use the findings of this review to conclude whether PM from some emission sources are clearly and consistently associated with worse health outcomes than PM from other sources.

Despite the extensive epidemiological studies that have recently examined source-specific PM, the main conclusion from this review of published research is that we are still not in a position to rank the harm that exposure to particles from different common emission sources has on human health.

## 2. A Comparison of the Health Effects Associated with Five Different Source-Specific PM

### 2.1. Traffic

Traffic generates airborne particles via exhaust emissions from fuel combustion, as well as the resuspension of non-exhaust PM from road, tyre, and brake wear. Non-exhaust PM is predominantly in the coarse fraction between 2.5 and 10 µm in diameter and is an important source of trace metals in PM in urban environments [46]. Particles from vehicle exhaust constitute the major source of ultrafine particles, <0.1 µm in diameter (PM_0.1_), in urban environments [47]. Traffic-generated PM includes secondary PM formed from hot exhaust gases (carbon dioxide, carbon monoxide, hydrocarbons, and nitrogen oxides) expelled from vehicle tailpipes. These gases include air toxics such as benzene, formaldehyde, acetaldehyde, and 1,3-butadiene, which can cause adverse health effects [46].

Traffic is a significant contributor to urban air pollution and the health effects of exposure to traffic-related air pollution (TRAP) have been extensively reviewed [46,48]. An expert panel for the US Health Effects Institute concluded that while many health effects have been associated with exposure to TRAP, only the evidence related to the exacerbation of childhood asthma was sufficient to assign causality [46]. The panel categorized the evidence linking the onset of childhood asthma, respiratory symptoms, impaired lung function, all-cause mortality, and cardiovascular morbidity with exposure to TRAP, as ‘suggestive but not sufficient’ to infer causation. The US Environmental Protection Agency (EPA) has implicated TRAP as a risk factor for myocardial infarction and also concluded that associations between ambient PM_2.5_ and cardiovascular disease hospitalisations may be primarily due to particles from traffic [49]. Various toxicological and epidemiological studies implicate traffic-related PM as likely to be causal in the associations between TRAP and cardiovascular health effects [50].

As TRAP includes both the gaseous and particulate components of traffic emissions, in health effect studies of TRAP it can be difficult to discern which components (or mixtures) are responsible for specific health effects. Health effects such as reduced lung function in children [51], increased blood pressure [52], allergic sensitisation [53], and premature birth [38], have each been associated with exposure to TRAP, but not specifically associated with traffic-related PM. However, associations suggest that PM contributes to the health impacts of TRAP.

PM_2.5_ apportioned to traffic has been associated with all-cause, respiratory, and cardiovascular mortality, and daily hospital admissions for cardiovascular disease, stroke, and heart failure [54,55,56,57,58] (Table 2). In the *Harvard Six Cities* cohort the magnitude of the effect of exposure to traffic-related PM_2.5_ on daily mortality was greater than that for PM_2.5_ from coal combustion, crustal dust, or total PM_2.5_ mass [56,58]. In the multi-country study, *Air Pollution on Health: A European Approach Study (APHEA2)*, PM from areas with higher ambient nitrogen dioxide (a marker of traffic emissions) was associated with greater acute health effects, suggesting that PM emitted by traffic is more toxic than PM from other sources [59].

Single city studies in New York, United States and Seoul, South Korea have observed traffic-related PM_2.5_ to be associated with daily cardiovascular hospital admissions and respiratory mortality, respectively [54,58], associations that were not observed with PM_2.5_ from either crustal dust or coal-fired power stations.

Findings from toxicological studies conducted within the NPACT project suggest that PM_2.5_ from vehicle exhaust emissions has greater cardiovascular toxicity than non-exhaust PM_2.5_, however epidemiological investigations within the project were inconclusive in their support of the working hypothesis that combustion-related PM_2.5_ components are more toxic than non-combustion components [60]. Long-term exposure to transition metals in PM_2.5_ (non-exhaust traffic emissions are a major source) was associated with increases in inflammatory markers in an investigation within the TRANSPHORM and ESCAPE projects [36].

Particles from traffic have high oxidative potential, possibly due to metals arising from engine and brake abrasion. Some studies, but not all, have demonstrated that as traffic density increases, the capacity of roadside PM to generate tissue-damaging reactive oxygen species increases [61]. PM from high-traffic density sites has been shown to be more toxic to animals than PM from other locations [62,63]. Cytotoxicity and pulmonary inflammation observed in those animal studies provide mechanistic support for some of the epidemiological observations.

Despite the many investigations of the health effects of PM from traffic emissions that have been conducted in a variety of settings and utilising different methods, the World Health Organisation (WHO) *Review of evidence on health aspects of air pollution (REVIHAAP)*, concluded that because of limited data and the large variability in outcomes and source characterisation methods among studies, the harmfulness of traffic-related PM cannot be ranked relative to other particle sources [48]. Nevertheless, source apportionment and toxicity studies suggest that traffic-related PM may be more harmful than PM from some other sources, including coal-fired power stations and crustal dust.

### 2.2. Coal-Fired Power Stations

Ambient PM resulting from coal combustion in power stations includes both primary PM emitted directly from power station smoke stacks and secondary PM formed in the atmosphere from emissions of sulphur and nitrogen oxide gases. The particles are primarily PM_2.5_, and since coal-fired power stations are a large source of sulphur dioxide, ambient concentrations of PM_2.5_-sulphate are considered a reliable marker of coal-fired power station emissions. In many industrialised countries, PM from coal-fired power stations is a significant contributor to total ambient PM levels, and the health effects of exposure to PM from this source have been widely investigated.

In a study conducted in 75 US cities over six years, two-day averaged PM_2.5_ with a high sulphur content was associated with both all-cause and respiratory mortality but not cardiovascular mortality [19]. Other epidemiological studies have observed mixed results for associations between PM_2.5_ from coal combustion and adverse cardiovascular disease outcomes [64,65,66], while several studies have observed associations between PM_2.5_-sulphate and daily respiratory disease hospitalisations [65,66,67]. A meta-analysis of ten single-city time-series studies (eight from North America and two from Europe) determined that PM_2.5_-sulphate was significantly associated with all-cause mortality [34].

The US EPA *Integrated Science Assessment for PM* concluded that while short-term exposure to secondary sulphate and PM_2.5_ from power stations has been associated with cardiovascular and respiratory health effects, the evidence is neither consistent nor robust [49]. However, the UK Government’s *Committee on the Medical Effects of Air Pollutants (COMEAP)* concluded that ‘there is reasonably strong evidence of a positive effect’ of short-term exposure to PM-sulphate on both cardiovascular and respiratory disease outcomes and an especially strong effect on mortality [68]. A WHO review noted that *secondary inorganic particulate air pollution*, which is typically characterised by PM_2.5_-sulphate, has been associated with cardio-respiratory health effects in most studies published since 2005 [48]. Secondary inorganic particulate air pollution includes PM not only from coal combustion, but also from oil combustion (including vehicle exhausts). A shortcoming of using ambient concentrations of a single species such as sulphate to indicate source-specific emissions, is that the species is rarely derived from a single source. An alternative approach by which to identify ambient PM derived from a single emission source is to use a number of chemical and physical properties that are typical of particles from that emission source, a method known as *source apportionment*.

Source apportionment studies have reported associations between exposure to PM from coal-fired power station emissions and all-cause, cardiovascular, pneumonia, and lung cancer mortality [57,58,69,70,71] (Table 2). A time-series analysis of source apportioned PM in Atlanta, Georgia, observed that hospital emergency department visits for respiratory disease were associated with exposure to PM_2.5_ from coal-fired power station emissions but not PM_2.5_ from gasoline exhaust, diesel exhaust, or wood smoke [72]. In a similar study in Washington DC, daily mortality was associated with PM_2.5_ from coal-fired power stations but not PM_2.5_ apportioned to traffic, soil, or wood smoke [69]. In Phoenix, Arizona, across six different emission sources, PM_2.5_ from coal-fired power stations and traffic had the largest effects on daily cardiovascular mortality although the effects were not consistent across different methods of source apportionment [70]. Nevertheless, the observation that PM_2.5_ from coal-fired power stations and traffic had the largest effect on cardiovascular mortality is consistent with findings from Barcelona, Spain [57]. Results of an investigation of source-specific PM_2.5_ and mortality in the *American Cancer Society’s Cancer Prevention II Study* cohort suggest that long-term exposure to PM_2.5_ from combustion sources, especially coal, may explain most of the mortality effects seen in earlier studies of that cohort, particularly in relation to ischemic heart disease [71].

Source apportionment studies have also reported a greater magnitude of health effects associated with PM_2.5_ from coal-fired power stations compared to total PM_2.5_ mass [57,72]. One difficulty with making such comparisons in locations where coal-fired power station emissions contribute significantly to ambient PM_2.5_ is that PM_2.5_ from this source is highly correlated with total ambient PM_2.5_.

There has been minimal investigation of the toxicity of PM from coal-fired power stations and the mechanisms of biological effects specific to these particles are largely unknown. The unifying hypothesis for the toxicity of combustion-derived particles in general is that the inhalation of these particles causes inflammation via oxidative stress and activation of redox-sensitive transcription factors, which leads to adverse health effects [73]. In support of this hypothesis applying to coal-fired power station emissions, elevated levels of blood markers of oxidative stress and oxidative DNA damage have been found in people who spent time in close proximity to coal-fired power stations [74,75]. Results from a series of toxicological studies designed to evaluate the health effects from various coal-fired power station emission scenarios suggest that the toxicity of PM from coal-fired power stations increases as the particles are atmospherically transformed (*photochemically aged*) [76].

In summary, there is considerable evidence that exposure to PM derived from coal-fired power station emissions, either emitted directly from power station stacks or generated during secondary formation in the atmosphere from emitted gases, is associated with adverse effects on health, particularly cardiovascular health. There is some evidence that exposure to PM from coal-fired power stations is associated with greater adverse health effects compared to exposure to total ambient PM mass and other sources of PM such as crustal dust and wood smoke.

### 2.3. Diesel Exhaust

Diesel exhaust particles from modern, optimal combustion engines are primarily PM_2.5_, a considerable component of which are PM_0.1_. They are highly complex particles with a core of elemental carbon and adsorbed organic compounds, as well as small amounts of sulphate, nitrate, metals, and many trace elements [77].

The study of the health effects of diesel exhaust PM is complicated by the fact that diesel exhaust PM varies in chemical composition and size according to engine type (heavy-duty, light-duty, method of fuel injection), engine operating conditions (idle, accelerating, decelerating), and fuel formulations (high/low sulphur fuel, petroleum-based diesel, biodiesel). It is unclear how these differences change toxicity [48,78]. Furthermore, the atmospheric lifetime of diesel exhaust PM ranges from minutes to several days and there is limited information about the physical and chemical transformation of these particles in the atmosphere, and what the overall toxicological consequences of these transformations are [79]. There is some indirect evidence that diesel exhaust particles may impact health to a greater extent as they age in the atmosphere [80].

Reviews of the health effects of diesel exhaust PM conducted around the turn of the century concluded that diesel exhaust PM is toxic to humans and that short-term exposure to diesel exhaust PM is associated with respiratory health effects such as allergic inflammation and symptoms typical of asthma, while long-term exposure is associated with enhanced lung cancer risk [79,81]. Those reviews considered short-term exposures in chamber studies, occupational epidemiology, exposures to populations near roadways, and toxicological studies in research animals. A more recent analysis of pooled data from 11 case-control occupational epidemiology studies conducted in Europe and Canada found that cumulative diesel exhaust exposure was associated with an increased lung cancer risk [82], and in 2012 the International Agency for Research on Cancer (IARC) classified diesel engine exhaust as ‘carcinogenic to humans’ based on findings from occupational epidemiological studies and toxicological investigations conducted in research animals [83]. The occupational epidemiological studies on which the IARC conclusions were based were limited by a general lack of objective measure of diesel exposure. Recent cohort and nested case-control studies in 12,000 US mine workers, which included PM measurements in their exposure assessment, observed that exposure to diesel exhaust PM was associated with lung and oesophageal cancer mortality [84,85]. However, not all reports have found such links between diesel exposure and cancer. A systematic review published in 2014 of 42 cohort and 32 case-control studies did not find a clear relationship between diesel exposure and lung cancer [86]. A literature review published in 2012 concluded that the occupational epidemiological evidence was inadequate to confirm a link between diesel exhaust exposure and lung cancer, and suggested that weak exposure–response associations could be explained by bias, confounding, chance, or exposure misclassification [87].

Due to the difficulty in distinguishing PM derived from diesel exhaust from PM arising from other emission sources, most epidemiological studies have not assessed the effects of exposure to ambient diesel exhaust PM. For example, although elemental carbon is present in much higher quantities in diesel emissions than in gasoline emissions, a unique tracer to separate gasoline from diesel emissions was not available in the NPACT study of mortality and long-term exposure to PM_2.5_ and its components in the *American Cancer Society’s Cancer Prevention Study II* cohort [71]. Nevertheless, the finding in the NPACT study that the ischaemic heart disease mortality risk estimate for elemental carbon was stronger than that for the ‘traffic’ source category suggests that diesel PM_2.5_ may have a greater effect on this outcome than gasoline PM_2.5_ emissions. Other toxicological and epidemiological NPACT studies support the notion of PM_2.5_ emissions with a high elemental carbon content contributing to cardiovascular health effects [88]. Further support for exposure to diesel exhaust PM being associated with cardiovascular effects comes from studies that have differentiated ambient source-specific PM on the basis of particle characteristics and correlation patterns. These studies have reported associations between exposure to diesel exhaust PM and daily hospital admissions and emergency room visits for cardiovascular disease [72,89].

It is noteworthy that the IARC deliberately excluded evidence from non-occupational exposure of diesel exhaust in their assessment of the carcinogenicity of diesel exhaust emissions because of the difficulty in assessing the contribution to cancer risk of diesel exhaust in ambient air [83]. Due to the technical difficulty of assigning exposure specifically to PM from diesel emissions, there are limited source apportionment studies comparing the health effects associated with PM from diesel emissions to PM from other sources. A source apportionment study conducted in Seoul, South Korea, found that exposure to PM_2.5_ from diesel emissions was associated with a greater daily respiratory mortality risk, but not total or cardiovascular mortality risk, compared to PM_2.5_ apportioned to eight other emission sources [54]. A study in Atlanta, Georgia, observed that by two different source apportionment methods, diesel exhaust PM_2.5_ (along with PM_2.5_ from gasoline exhaust and wood smoke emissions) was associated with daily emergency department cardiovascular visits whereas PM_2.5_ apportioned to soil and coal-fired power stations were not [72].

The majority of evidence indicating the potential for diesel exhaust PM to cause health effects has come from human chamber studies and studies in research animals. Controlled exposures of humans to diesel exhaust have resulted in various cardiovascular changes indicative of increased acute coronary event risk, mild constriction and inflammation of lung airways, nose and throat irritation, and changes in lung function [48,49,90]. In most chamber studies, no distinction was made as to which components of diesel exhaust (particles or gases) were responsible for the observed effects. There is some toxicological evidence that the particle component of diesel exhaust is responsible for observed cardiovascular effects [91,92].

Many research animal studies support the biological plausibility of the health effects observed in humans exposed to diesel exhaust [49,83,90,93,94,95]. As with PM from other sources, it is thought that oxidative stress underpins the mechanism by which diesel exhaust causes health effects, and the effects of diesel exhaust PM may be accentuated in individuals with conditions associated with oxidative stress, such as diabetes and obesity [96,97]. Diesel exhaust PM has also been shown to enhance susceptibility to infection and increase the atopic response to allergens [98,99,100]. Exposure of pregnant mice to diesel exhaust PM has been found to affect the central nervous and immune systems of offspring, as well as their susceptibility to asthma and heart failure [101,102,103]. However, there is no evidence of inherited health effects from exposure to diesel exhaust at levels that are typical of ambient environments.

Overall, data convincingly demonstrate that diesel exhaust PM exerts effects on physiological endpoints with relevance to adverse health effects. However, the health impacts of long-term exposure to concentrations typical of ambient environments remain unknown and the potency of diesel exhaust PM relative to PM from other sources is unclear.

### 2.4. Domestic Wood Combustion Heaters

Domestic wood combustion heaters can significantly contribute to ambient PM in locations with cold or moderate winters [104,105,106,107,108]. Respiratory symptoms and exacerbations, particular among children, have been associated with elevated concentrations of ambient PM in wood-burning communities [109,110,111,112,113,114]. Wood combustion PM emissions appear to have less of an impact on cardiovascular health [111,112,115], although a source apportionment study in Atlanta, Georgia found daily wood smoke PM_2.5_ to be associated with hospital emergency department visits for cardiovascular disease but not respiratory disease [72]. That study observed the effect of wood smoke PM_2.5_ on daily cardiovascular emergency department visits to be similar in magnitude to that of gasoline and diesel PM_2.5_. A source apportionment study from Copenhagen, Denmark observed that PM_10_ derived from biomass (which in Copenhagen is primarily from wood burning) had a stronger association with daily respiratory hospital admissions than did PM_10_ apportioned to traffic [115], however other source apportionment studies suggest differently, with biomass/wood smoke generally having less of an impact on adverse health outcomes than PM from other combustion sources [54,69,70,71] (Table 2). It is important to note that the methods used to apportion PM in those studies mean that the smoke particles are not attributable specifically to wood heaters. The relative health impacts of PM emitted from wood combustion heaters compared to ambient PM from other sources remains unclear.

Toxicological studies have provided biological plausibility for wood smoke affecting respiratory health via compromise of lung immune defence [112,116]. However, chronic exposure of research animals to concentrations of wood smoke relevant to ambient conditions has only resulted in mild airway inflammation, and had minimal effect on lung bacterial clearance [117,118]. Wood smoke contains compounds such as polycyclic aromatic hydrocarbons that have carcinogenic properties, and there is evidence that indoor exposure to wood smoke increases cancer risk [119]. However, the cancer risk associated with exposure to ambient wood smoke is unknown.

### 2.5. Crustal Dust

Crustal dust is a product of wind erosion, and most prevalent in arid and semi-arid climates. Primarily because they are an easily identifiable source of crustal dust, most studies of the health effects of crustal dust have made use of dust storms, events that can result in extremely high concentrations of PM air pollution.

Dust storm events have been associated with a variety of health effects [120]. Increases in all-cause mortality have been observed during dust storms in Australia [121], Seoul and other South Korean cities [122,123], and Taipei [124]. Most investigations suggest that exposure to high levels of crustal dust increases cardiovascular mortality [120], however daily cardiovascular hospital admissions are not usually associated with crustal dust [49,125,126,127,128,129,130,131].

Dust storms have been associated with increased respiratory disease presentations to hospital emergency rooms [127], exacerbations of asthma in adults [125,132,133,134] and children [135,136], and increased hospitalisations for COPD [137,138]. However, dust storms have generally not been associated with increases in respiratory mortality [123,124,139,140]. An exception, in Italy, Saharan dust storms were associated with respiratory mortality in people 75 years of age and older [141].

Asian dust storms have been associated with an increase in hospital admissions for pneumonia in Taipei [142], but not in other locations [125,138]. The incidence of pneumonia has also been associated with exposure to dust storms in the US, Russia, and the Middle East [143]. It is not clear how dust storms increase pneumonia, which is predominantly caused by viral or bacterial infection. Inhalation of crustal dust may suppress the immune response and allow commensal bacteria to cause disease, or pathogens transported on desert dust particles may be responsible for initiating disease [143]. Other infectious diseases that have been associated with dust storms include coccidiomycosis (fungal), meningococcal meningitis, and conjunctivitis [144,145,146].

Source apportionment studies have investigated the effects of exposure to crustal dust outside of dust storm events. Although sometimes associated with respiratory symptoms and cardiovascular effects, PM apportioned to soil or crustal dust (outside of dust storms) has generally not been associated with adverse health effects, suggesting that these particles are less toxic than PM from other sources [54,55,58,69,70,71,72] (Table 2). Crustal dust particles are clearly not benign. These particles induce an oxidative stress response and provoke inflammatory responses in animal lungs [147,148], toxicological effects that are comparable to effects elicited by PM from other sources. As well as containing components of the Earth’s crust, dust particles contain material picked up from industrialised and agricultural areas [149,150]. Anthropogenic metals in Asian dust storm particles have been associated with reduced lung function in children in Seoul [151]. It is likely that constituents acquired during transport contribute to the toxicity of dust particles.

### 2.6. Comparison of the Effect on All-Cause and Cardiovascular Mortality of Increases in Different Source-Specific PM_2.5_

**Figure 2:**
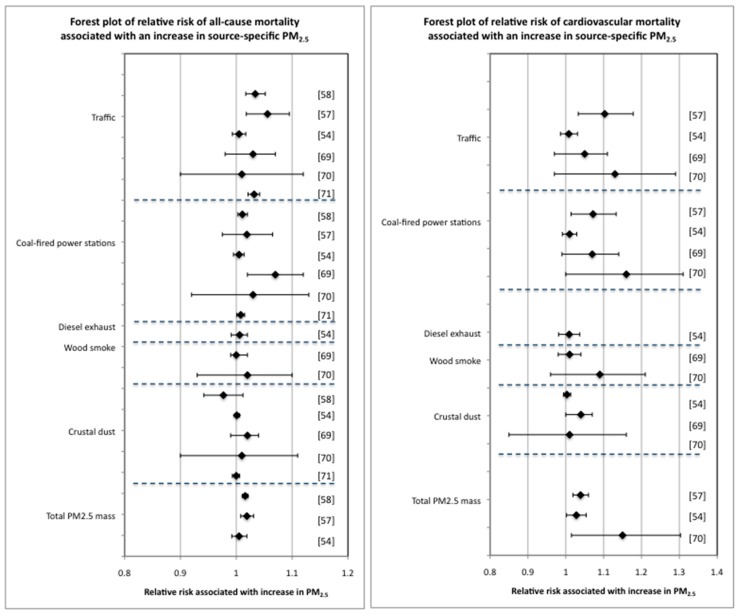
Forest plots of the change in all-cause and cardiovascular risk associated with increases in source-specific PM_2.5._ The data in these forest plots are from Table 2. The reference numbers applicable to the different data are shown.

The comparative effects of increases in source-specific PM_2.5_ on all-cause and cardiovascular mortality (from the source apportionment studies in Table 2) are shown in forest plots in Figure 2. The forest plots indicate that while there is no clear hierarchy in the impact that PM_2.5_ from different emission sources has on mortality risk, there is a suggestion that PM_2.5_ from traffic and coal-fired power stations have a greater mortality impact, especially in relation to cardiovascular diseases.

## 3. Discussion—Challenges in Differentiating Health Effects Associated with Exposure to PM from Different Emission Sources

There are many potential reasons why the comparative toxicity of source-specific PM is not well understood despite large research programs investigating this issue. Firstly, quantifying source-specific PM in ambient air, which contains a complex and dynamic mix of air pollutants, is a significant challenge. For example, source apportionment, the process of attributing ambient PM to emission sources based on particle composition, is dependent on which constituents of particles are used to assign PM to a source, and this technique is not capable of assessing the effects of interactions of different particles in particle mixtures [152]. Limitations in existing statistical methods are at least partially responsible for the lack of information on joint effects, an issue that Health Effects Institute research programs are attempting to address [152]. Secondly, a number of different methods of source apportionment and receptor modeling have been used in air pollution epidemiology [153], making the comparison of results between studies problematic. Thirdly, the heterogeneity of populations, emission sources, and air pollution mixtures between different locations mean that study results can vary significantly between cohorts [154]. Fourthly, PM emissions from different anthropogenic sources are often highly correlated, making it difficult to determine the contribution of source-specific PM to an associated health effect.

An alternative approach to source apportionment that has been used in recent analyses of ESCAPE cohorts is to use land use regression models to estimate exposure to elemental and molecular components of PM [36,37,154,155]. These studies offer insights into likely source candidates for observed health effects. However, individual components of PM might represent one or more sources, and estimated component concentrations might reflect different sources between cohorts because of different predictor variables included in the land use regression models [36].

## 4. Conclusions on the Comparative Health Effects of Source-Specific PM Air Pollution from This Review

The evidence of health effects associated with source-specific PM does not indicate a clear ‘hierarchy’ of harmfulness for PM from different emission sources (Figure 2 and Table 3). PM from different sources may differ in population health impact due to variation in the extent of exposure, but for equivalent exposures it is not clear which particles from which emission sources are most detrimental to population health. No source-specific PM or PM components have been shown unequivocally to not be associated with health effects [156], however despite a proliferation in comparative studies there is little consistency among findings on which to form a consensus on which sources generate PM with the greatest potential to affect health. There is at least some similarity in research findings to suggest that the health effects of exposure to PM from traffic and coal-fired power stations may be greater than the effects of exposure to PM from other sources [56,57,58,69,70,71,72]. However, more studies are required to establish quantitative and qualitative differences in the health effects of PM from these different sources.

The health effects of PM air pollution, including health effects attributable to PM from specific emission sources, is a large field of investigation. In attempting to compare evidence of health effects related to exposure to PM from five common emission sources (traffic, coal-fired power stations, diesel exhaust, domestic wood combustion heaters, and crustal dust), it was not possible for this article to refer to all of the published studies that have investigated health effects associated with PM from these emission sources. Investigation of traffic alone has resulted in hundreds of publications on the health effects of PM from this emission source. However, major investigations of the comparative health effects of PM from the different emission sources have been included, and we think that it is unlikely that sufficient comparative studies have been excluded that would have changed our general conclusion on the comparative harmfulness of PM from the different sources.

This review has a focus on epidemiological studies that compare different PM emission sources. However, toxicological studies in humans and research animals will help to inform the likely health effects of exposure to source-specific PM. Unlike epidemiological studies, toxicological studies have the advantage of being able to associate effects with precise exposures. Their disadvantage is that they lack the “real-world” conditions within which population relevant exposures occur. It is likely that no one study type will be able to determine the relative health effects of PM from different sources, and that only the cumulative evidence from a range of study types, each with different strengths and limitations, will provide some clarity in this area.

Research described in this review has linked a variety of health effects to source-specific PM. However, more advanced approaches to modeling, measurement, and statistics will be required to more precisely quantify health effects attributable to exposures in the multi-pollutant atmosphere. Determination of the relative health effects of different source-specific PM will help to inform policy and regulatory strategies to reduce the public health burden of ambient PM. Enhanced understanding of these relative health effects offer the potential for better targeted public health protection than the current recommended practice of minimising exposure to total PM mass, regardless of the source.

## Figures and Tables

**Table 1:** Conclusions from previous reviews of the differences in the health effects of different components and sources of PM air pollution.

Reference	Study Conclusions in Relation to Health Effects of Source-Specific PM Air Pollution
[38]	The black carbon, for which vehicles and particularly diesel vehicles are a major source in urban areas, in PM might make PM from those sources the most harmful. The relative toxicity of wood smoke compared with vehicle exhaust emissions is unclear.
[29]	Current evidence does not allow a precise differentiation to be made as to which constituents or sources of PM are most closely related to specific health outcomes. However, three components, black carbon, secondary organic aerosols, and secondary inorganic aerosols may be important contributors to PM toxicity.
[39]	Current knowledge does not allow precise quantification or definitive ranking of the health effects of PM from different sources. However, some results suggest that a range of serious health effects are more consistently associated with traffic-related PM and specific metals and elemental carbon in PM.
[40]	There is a lack of information by which to differentiate the toxicity of different components of PM.
[41]	Evidence suggests that carbon components and several metals in PM are associated with health effects however it is unclear whether these components are responsible for health impacts or they are surrogates for other pollutants.
[31]	Cardiovascular health effects may be associated with PM_2.5_ from crustal or combustion sources, including traffic, but at this time, no consistent relationships have emerged. Collective evidence has not yet isolated factors or sources that would be closely and unequivocally related to specific health outcomes.
[42]	There is evidence that metals within PM affect health but considerable uncertainties about causality remain.
[43]	Evidence relating to the toxicity of inorganic components of PM_2.5_ is not consistent. Crustal components of PM_2.5_ are not likely, by themselves, to be a significant health risk.
[44]	Public health will likely be better protected by reduction of various vehicular emissions than by regulation of total PM_2.5_ mass as if all PM_2.5_ is equitoxic. However, the knowledge base is incomplete.
[45]	There is little support for the idea that any single major or trace component of PM is responsible for the adverse health effects of PM.

**Table 2:** Source-apportionment studie s that compared the health effects of different source-specific PM_2.5_ within studies.

Ref.	Method Used to Identify Source-Specific PM	Health Outcomes Investigated	Relative Risk Associated with an Increase in PM_2.5_ (95% Confidence Interval) ^1^					
Source			Traffic	Coal-Fired Power Stations (Secondary Sulphate)	Diesel Exhaust	Wood Smoke	Crustal Dust (Soil)	All (Total Mass)
[56]	Factor analysis to identify up to 5 common factors from 15 specified elements	Daily all-cause mortality per 10 µg/m^3^ increase in PM_2.5_	1.03 (no CI’s)					1.05 (no CI’s)
[58]	Factor analysis to identify up to 5 common factors from 15 specified elements	Daily all-cause mortality per 10 µg/m^3^ increase in PM_2.5_	1.034 (1.017–1.052)	1.011 (1.003–1.020)			0.977 (0.942–1.012)	1.016 (1.011–1.021)
[55]	Positive matrix factorization	Daily cardiovascular and respiratory hospital admissions per 5–95th percentile increase in PM_2.5_	1.04 (1.01–1.08) ^2^ (cardiovascular) 1.01 (0.97–1.06) ^2^ (respiratory)	1.01 (0.97–1.05)^2^ (cardiovascular) 1.03 (0.97–1.09) ^2^ (respiratory)			1.00 (0.95–1.04) ^2^ (cardiovascular) 1.02 (0.96–1.09) ^2^ (respiratory)	1.01 (0.98–1.05) ^2^ (cardiovascular) 1.05 (1.00–1.10) ^2^ (respiratory)
[57]	Positive matrix factorization	Daily all-cause and cardiovascular mortality per IQR increase in PM_2.5_	1.056 (1.018–1.095) (all-cause) 1.103 (1.033–1.178) (cardiovascular)	1.019 (0.975–1.065) (all-cause) 1.072 (1.014–1.133) (cardiovascular)				1.019 (1.008–1.031) (all-cause) 1.039 (1.019–1.060) (cardiovascular)
[54]	Positive matrix factorization	Daily all-cause, cardiovascular and respiratory mortality per IQR increase in PM_2.5_	1.005 (0.993–1.017) (all-cause) 1.008 (0.986–1.031) (cardiovascular) 1.055 (1.005–1.107) (respiratory)	1.005 (0.995–1.014) (all-cause) 1.010 (0.991–1.029) (cardiovascular) 1.021 (0.983–1.061) (respiratory)	1.006 (0.991–1.020) (all-cause) 1.009 (0.981–1.037) (cardiovascular) 1.067 (1.002–1.137) (respiratory)		1.001 (0.997–1.006) (all-cause) 1.003 (0.994–1.013) (cardiovascular) 1.016 (0.997–1.035) (respiratory)	1.005 (0.992–1.019) (all-cause) 1.028 (1.002–1.054) (cardiovascular) 1.021 (0.972–1.071) (respiratory)
[72]	Positive matrix factorization	Daily cardiovascular and respiratory emergency department (ED) visits per IQR increase in PM_2.5_	1.022 (1.012–1.032) ^2^ (cardiovascular) 0.999 (0.993–1.007)^2^ (respiratory)	1.004 (0.992–1.021) ^2^ (cardiovascular) 1.015 (1.002–1.028) ^2^ (respiratory)	1.030 (1.017–1.039) ^2^ (cardiovascular) 0.997 (0.991–1.005) ^2^ (respiratory)	1.029 (1.018–1.037) ^2^ (cardiovascular) 0.999 (0.993–1.006) ^2^ (respiratory)	1.005 (0.998–1.012) ^2^ (cardiovascular) 0.998 (0.993–1.003) ^2^ (respiratory)	1.025 (1.008–1.041) ^2^ (cardiovascular) 1.007 (0.996–1.019) ^2^ (respiratory)
[69]	Various multivariate factor analysis based receptor models	Daily all-cause and cardiovascular mortality per 5–95th percentile increase in PM_2.5_	1.03 (0.98–1.07) ^2^ (all-cause) 1.05 (0.97–1.11) ^2^ (cardiovascular)	1.07 (1.02–1.12) ^2^ (all-cause) 1.07 (0.99–1.14) ^2^ (cardiovascular)		1.00 (0.99–1.02) ^2^ (all-cause) 1.01 (0.98–1.04) ^2^ (cardiovascular)	1.02 (0.99–1.04) ^2^ (all-cause) 1.04 (1.00–1.07) ^2^ (cardiovascular)	
[70]	Various multivariate factor analysis based receptor models	Daily all-cause and cardiovascular mortality per 5–95th percentile increase in PM_2.5_	1.01 (0.90–1.12) ^2^ (all-cause) 1.13 (0.97–1.29) ^2^ (cardiovascular)	1.03 (0.92–1.13) ^2^ (all-cause) 1.16 (1.00–1.31) ^2^ (cardiovascular)		1.02 (0.93–1.10) ^2^ (all-cause) 1.09 (0.96–1.21) ^2^ (cardiovascular)	1.01 (0.90–1.11) ^2^ (all-cause) 1.01 (0.85–1.16) ^2^ (cardiovascular)	Not reported (all-cause) 1.150 (1.015–1.303) (cardiovascular)
[71]	Multivariate factor analysis of elemental data with source modeling	All-cause, ischemic heart disease (IHD) and respiratory mortality per IQR increase in PM_2.5_	1.032 (1.021–1.042) ^2^ (all-cause) 1.013 (0.987–1.039) ^2^ (IHD) 1.09 (1.05–1.13) ^2^ (respiratory)	1.008 (1.001–1.015) ^2^ (all-cause) 1.042 (1.024–1.060) ^2^ (IHD) 0.95 (0.92–0.97) ^2^ (respiratory)			1.000 (0.993–1.006) ^2^ (all-cause) 1.000 (0.986–1.012) ^2^ (IHD) 1.02 (1.00–1.04) ^2^ (respiratory)	

^1^ Results are only shown for the emission sources covered by this review. Where a study examined the change in health outcome risk over several days of lag after the exposure to PM_2.5_, the result that is shown corresponds to the lag for which there was the maximum increase in the health outcome. ^2^ Values are approximations read from figures in the respective references.

**Table 3:** Summary of PM emission sources and reported health and physiological/toxicity effects (physiological/toxicity effects includes animal studies).

Emission Source	Health Risk and Reference
**Traffic**	
Total traffic-related air pollution (TRAP)	exacerbation and onset of childhood asthma, respiratory symptoms, impaired lung function, all-cause mortality, cardiovascular morbidity [46]
	myocardial infarction [49]
	reduced lung function in children [51]
	increased blood pressure [52]
	allergic sensitization [53]
	premature birth [38]
Specifically traffic PM	all-cause, respiratory and cardiovascular mortality, cardiovascular, stroke and heart failure morbidity [54,55,56,57,58,70,71,72]
	cardiovascular toxicity and various cardiovascular effects [50,60]
	cytotoxicity, pulmonary inflammation [62,63]
**Coal-fired power stations**	all-cause, cardiovascular, respiratory, ischaemic heart disease, pneumonia, lung cancer mortality [19,34,57,58,69,70,71]
	respiratory morbidity [48,49,65,66,67,68,72]
	cardiovascular morbidity [48,49,68]
**Diesel exhaust**	respiratory mortality [54]
	lung and oesophageal cancer mortality [84,85]
	allergic inflammation, asthma symptoms, lung cancer [79,81,82,83]
	cardiovascular morbidity [72,89]
	cardiovascular changes indicative of increased coronary event risk, changes in lung function, nose and throat irritation [48,49,90]
	atopy and susceptibility to infection [98,99,100]
	effects on offspring from exposure during pregnancy [101,102,103]
**Domestic wood combustion heaters** (studies of outdoor exposure to heater emissions)	respiratory symptoms and exacerbations [109,110,111,112,113,114]
	cardiovascular morbidity [72]
	respiratory morbidity [115]
	compromised lung immunity, airway inflammation [112,116,117,118]
**Crustal dust**	all-cause and cardiovascular mortality [120,121,122,123,124]
	respiratory mortality(>75 years of age) [141]
	respiratory and COPD morbidity [127,137,138]
	asthma exacerbation [125,132,133,134,135,136]
	reduced lung function in children [151]
	pneumonia [142,143]
	lung inflammation [147,148]
	infectious disease [144,145,146]
